# Non-invasive monitoring of oxygen delivery in acutely ill patients: new frontiers

**DOI:** 10.1186/s13613-015-0067-7

**Published:** 2015-09-17

**Authors:** Azriel Perel

**Affiliations:** Anesthesiology and Intensive Care, Sheba Medical Center, Tel Aviv University, 52621 Tel Aviv, Israel

**Keywords:** Oxygenation, Pulse oximetry, Blood transfusion, Monitoring, Fluid responsiveness, Plethysmographic variation index (PVI)

## Abstract

Hypovolemia, anemia and hypoxemia may cause critical deterioration in the oxygen delivery (DO_2_). Their early detection followed by a prompt and appropriate intervention is a cornerstone in the care of critically ill patients. And yet, the remedies for these life-threatening conditions, namely fluids, blood and oxygen, have to be carefully titrated as they are all associated with severe side-effects when administered in excess. New technological developments enable us to monitor the components of DO_2_ in a continuous non-invasive manner via the sensor of the traditional pulse oximeter. The ability to better assess oxygenation, hemoglobin levels and fluid responsiveness continuously and simultaneously may be of great help in managing the DO_2_. The non-invasive nature of this technology may also extend the benefits of advanced monitoring to wider patient populations.

## The concept of oxygen delivery (DO_2_)

Normally, about 1000 ml of oxygen (O_2_) is delivered to the tissues each minute. This amount of O_2_ is termed ‘oxygen delivery’ (DO_2_). The DO_2_ is dependent on the cardiac output (CO) and on the arterial O_2_ content (CaO_2_) which is the amount of O_2_ that is carried by a 100 ml of arterial blood. Hence the DO_2_ is calculated by the multiplication of these two components—DO_2_ = CO × CaO_2_ × 10. In turn, the CaO_2_ is dependent on the hemoglobin (Hgb) concentration, the O_2_ saturation (SaO_2_) and the partial pressure of O_2_ (PaO_2_), as can be seen in the following formula—CaO_2_ = Hgb × 1.39 × SaO_2_ + (PaO_2_ × 0.0032).

Since the concept of DO_2_ conveniently summarizes the overall function of both circulation and oxygenation, it has been repeatedly used as a therapeutic ‘goal’ in both critically ill and high-risk surgical patients. For example, only recently it was shown that achievement of preoperative DO_2_ values in the postoperative phase was associated with less morbidity in patients undergoing high-risk elective surgery [1].

**Fig. 1:**
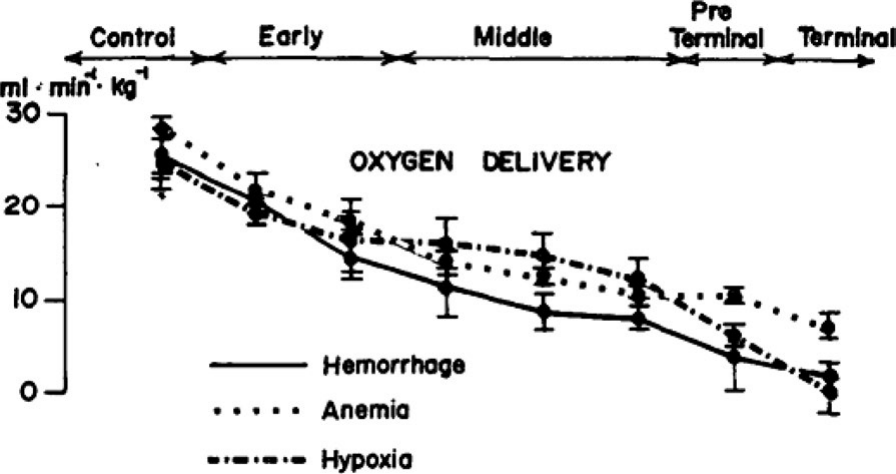
The effects of hypovolemia, anemia and hypoxia on the oxygen delivery. Sequential measurement of oxygen delivery in 30 dogs during controlled hypovolemia, normovolemic anemia and hypoxia. (Reproduced with permission from [2])

A deterioration in each of the components of the DO_2_ may lead to a life-threatening situation and death [2] (Fig. 1). Therefore, many of our therapeutic decisions are aimed at the prevention, early identification or timely improvement of hypovolemia, anemia and hypoxemia. While this may seem quite straightforward, fluids, blood and oxygen should be considered as drugs that need careful titration, since when administered in excess they may be associated with severe consequences. To complicate things further, there is an ever-going debate about how to best administer these potentially life-saving treatments.

What can be of great help in this uncertain environment is our improved ability to monitor the individual physiological variables that determine the DO_2_. This review attempts to describe some of the newly introduced technologies that enable us to do so, continuously and non-invasively, via the existing sensor of the pulse oximeter. However, like all other cardiorespiratory variables, these novel parameters have inherent limitations, inaccuracies and confounding factors. In addition, validation studies of these variables have been mostly carried out in the perioperative environment, and their validation in critically unstable patients is still lacking. In spite of these limitations, the non-invasive nature of these technologies and their potential to offer new insights in wider patient populations, represent true advancement in patient monitoring and safety.

## Non-invasive monitoring of oxygenation

### O_2_ saturation (SpO_2_)

The role of pulse oximetry in clinical anesthesia and in intensive care has evolved to the point where it is unlikely that we will ever be able to do without it [3]. Indeed the routine monitoring of SpO_2_ has greatly improved patient safety, as evidenced by the marked decrease in the proportion of malpractice claims due to respiratory events during anesthesia [4]. And yet, hypoxemia continues to occur commonly in the operating room [5] and in the post-anesthesia care unit (PACU) [6]. In the ICU SpO_2_ is often used to titrate the FiO_2_ (see later) but has also been recently suggested to play a role in the definition of ARDS. For the definition of ARDS in pediatric patients it is suggested that pulse oximetry-based criteria be used when PaO_2_ is unavailable, and that the oxygen saturation index (SpO_2_/FiO_2_) replace the PaO_2_/FiO_2_ ratio [7]. For the definition of ARDS in adults, it was recently suggested that the PaO_2_/FiO_2_ ratio should be calculated under standardized ventilator settings of PEEP (10 mmHg) and FiO_2_ (0.5), unless the SpO_2_ is below 88 % [8]. Obviously a robust SpO_2_ monitor is needed in such circumstances [9] which may often be also associated with decreased perfusion.

In view of its impact on patient safety we may want to consider using pulse oximetry in more instances and environments. For example, it was recently reported that hypoxemia is prevalent in patients on arrival to the PACU, and that in only few of these patients it could be identified clinically by anesthetists and nurses [10]. Considering the uncertainty about the deleterious effects of transient short-lasting hypoxemia, the authors advocate the routine use of pulse oximetry for patient transfer to the PACU. What about monitoring SpO_2_ in patients on the general ward? Analysis of signs and symptoms considered by nurses as indicators of patient deterioration on the general ward and a reason for ‘worry’ calls to rapid-response teams has revealed that they include, first and foremost, change in breathing and a decrease in SpO_2_ [11]. The introduction of a pulse oximetry and respiratory rate-based patient surveillance system (SafetyNet, Masimo, Irvine, CA, USA) with nursing alarm notification via wireless pager in a 36-bed orthopedic unit, resulted in a significant decrease in rescue events and in ICU transfers [12]. The recent PERISCOPE study has found that about 4.2 % of surgical patients develop postoperative respiratory failure (PRF) which carries an in-hospital mortality of 10.3 vs. 0.4 % in the rest of the patients [13]. The authors of this study conclude that high-risk surgical patients who may be prone to develop PRF, should be allocated to more experienced physicians, who can make full use of the range of available resources for their postoperative monitoring and treatment. We can therefore expect that in the future the monitoring of SpO_2_ will become more prevalent in the general wards of acute care hospitals.

And yet we have to remind ourselves the limitations of SpO_2_ monitoring, the main one being that pulse oximetry is a useful tool to assess ventilator abnormalities, but only in the absence of supplemental inspired oxygen. For example, severe hypoventilation that occurs when the inspired FiO_2_ is 0.3, may result in both the partial pressures of CO_2_ (PaCO_2_) and O_2_ (PaO_2_) values to be around 100 mmHg each, without any change in the SpO_2_ [14]. This limitation may have important safety consequences to patients who are recovering from anesthesia, undergoing procedural sedation or receive potent opioids. In a recent review of 357 acute pain claims from the Anesthesia Closed Claims Project database, 92 cases involved likely opioid-related respiratory depression [15]. The vast majority of these injuries occurred within 24 h of surgery, resulted in death or severe brain damage, and was judged as preventable with better monitoring and response. Thirty-three percent of these patients were monitored by pulse oximeters and 15 % were receiving oxygen [15]. It is obvious that a more effective mean of ventilatory monitoring, such as capnography or the monitoring of respiratory rate [16], should be used more frequently in all patients who are susceptible to develop respiratory depression, especially when they are being administered supplemental oxygen.

### Oxygen reserve index (ORI)

From the equation that describes the CaO_2_ it is obvious that the partial pressure of O_2_ (PaO_2_) does not contribute significantly to DO_2_. However, during acute anemia, increasing the PaO_2_ by hyperoxic ventilation establishes a highly available source of O_2_ that can be effectively utilized for tissue oxygenation [17]. More frequently, however, the PaO_2_ is used to assess the status of oxygenation during supplemental oxygen administration, where the SpO_2_ may no longer be informative. This requires blood gas sampling and analysis that is intermittent and delayed. Between invasive sampling, changes in PaO_2_ cannot be assessed and therefore unexpected hypoxia or unintended hyperoxia may go unnoticed.

**Fig. 2:**
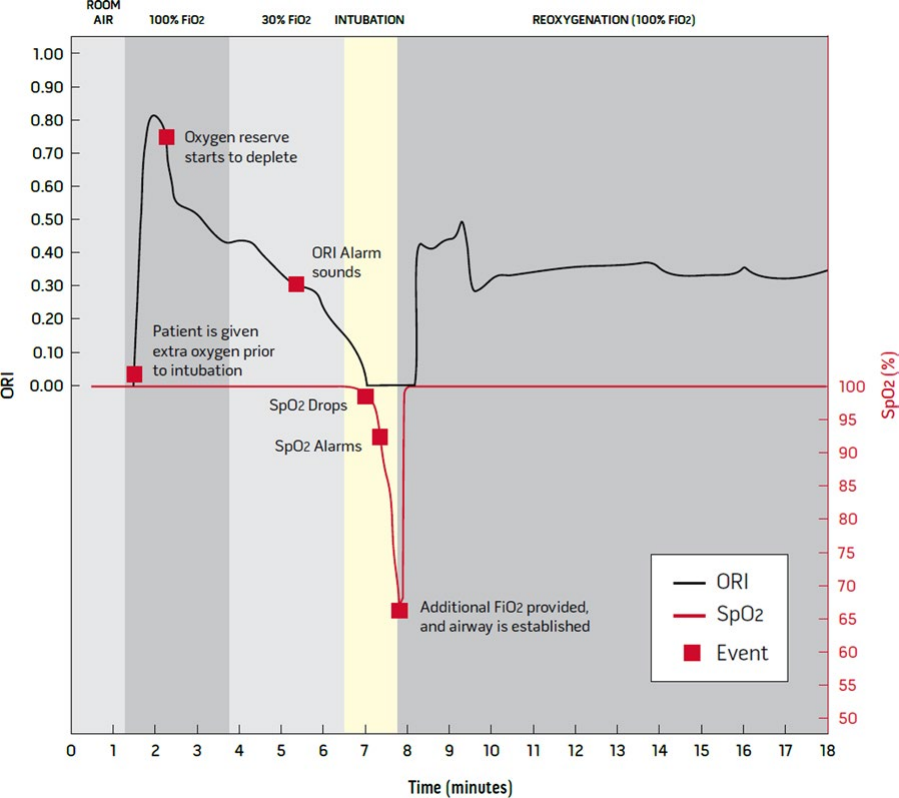
Example of the oxygen reserve index (ORI) during intubation in pediatric surgery. Note early increase of ORI during pre-oxygenation; decline in ORI triggering alarm before any change in SpO_2_ occurs; disappearance of ORI when SpO_2_ <100 %; re-appearance once SpO_2_ >100 %. (Figure provided by Masimo Corp. Irvine, CA, USA)

The oxygen reserve index (ORI) is a new feature of multiple wavelength pulse oximetry that provides real-time visibility to oxygenation status in the moderate hyperoxic range (PaO_2_ of approximately 100–200 mm Hg). The ORI is an “index” with a unit-less scale between 0.00 and 1.00 that can be trended and has optional alarms to notify clinicians of changes in a patient’s oxygen status. When utilized in conjunction with SpO_2_ monitoring the ORI may extend the visibility of a patient’s oxygen status into ranges previously unmonitored in this fashion. The ORI may make pre-oxygenation visible, may provide early warning when oxygenation deteriorates (Fig. 2), and may facilitate a more precise setting of the required FiO_2_ level.

The administration of high levels of inspired O_2_ before tracheal intubation (pre-oxygenation) is considered to be a routine practice as oxygen reserves are not always sufficient to prevent hypoxia during the duration of intubation [18]. The ORI may make this process visible, ensuring that the PaO_2_ is indeed rising in the presence of a constant maximal SpO_2_ level. The ORI may eventually become a performance indicator, attesting to the fact that pre-oxygenation has indeed been properly performed. Monitoring the ORI may be especially important in the presence of predictive risk factors for inadequate pre-oxygenation, which overlap with criteria predictive of difficult mask ventilation [18]. It may also be extremely important during pre-oxygenation before suctioning hypoxemic patients [19], during emergency rapid sequence induction [20], in obese patients [21], during intubation in the ICU [22], and especially in hypoxic patients who may require non-invasive ventilation before intubation [23].

The ORI may provide early warning of impending hypoxia before any changes in SpO_2_ occur (Fig. 2). In anesthetized pediatric patients the mean time (±SD) from the start of the ORI alarm to a decrease in SpO_2_ below 98 %and from SpO_2_ 98 to 90 % was 40 ± 523 and 52 ± 44 s, respectively (Szmuk P., et al. American Society of Anesthesiologists Annual Meeting, October 14, 2014. New Orleans; BOC12). In another recent trial in 103 anesthetized adult patients, ORI could be calculated ~91.5 % of monitored time, and was positively correlated with PaO_2_ values ≤240 mmHg but not with PaO_2_ >240 mmHg. PaO_2_ was ≥150 mmHg in 96.5 % of ORI >0.54, and was >100 mmHg for all ORI >0.24 (Applegate R, et al. S-377: International Anesthesia Research Society 2015 Annual Meeting, March 23, Honolulu, Hawaii). The early warning that the ORI provides before any decrease in SpO_2_ occurs may provide precious time for an earlier detection of the event and the provision of timely remedial measures.

Traditionally O_2_ is considered as a drug that may cause toxicity and current guidelines recommend using the lowest FiO_2_ possible. The importance of avoiding hyperoxemia has been recognized in both anesthetized [24] and in critically ill patients, especially those with ARDS [25]. However, it was recently proposed to consider resetting the target range for arterial oxygenation higher (85–110 mm Hg) as a potential strategy to improve the long-term outcomes (cognitive and physical impairment) of ARDS survivors [26]. Others claim that hyperoxia may be beneficial in septic shock and other acute clinical situations [27, 28]. Unintended hyperoxia seems to be quite common. According to a large Dutch study including 126,778 arterial blood gas samples from 5498 mechanically ventilated patients, hyperoxia (PaO_2_ was >120 mmHg) was found in 22 % of the samples but in only 25 % of these instances was the FiO_2_ decreased, implying that hyperoxia was accepted without adjustment in ventilator settings if FiO_2_ was 0.4 or lower [29]. Taking all the above into consideration, it is clear that the ORI may facilitate a more accurate control of the FiO_2_ in patients receiving O_2_ as it may identify and help prevent unwarranted hyperoxia. A spontaneous increase in the ORI may also be the first sign of pulmonary improvement in patients with respiratory failure.

## Non-invasive monitoring of hemoglobin (SpHb)

The other major component of the CaO_2_ besides the SpO_2_ is the hemoglobin (Hgb) concentration, which can now be measured via the pulse oximeter sensor by the new multiple wavelength pulse co-oximetry technology [30]. The continuous non-invasive measurement of hemoglobin (SpHb) allows a real-time identification of changes in Hb concentration between invasive blood sampling. This measurement may potentially prevent unnecessary transfusions and their associated complication, as well as identify undetected bleeding.

Red blood cell (RBC) transfusion is one of the most frequent procedures performed in acute care hospitals. However, transfusion practices are highly variable across countries, institutions, procedures and physicians [31, 32]. This variability most probably reflects a true controversy about the pros and cons of transfusion, but is also due in large part to lack of education and deeply ingrained ‘historical’ beliefs. Restrictive transfusion practices have been gaining a wider acceptance since the publication of the TRICC study in 1999, where the use of a Hb level of 7 mg/dL as a transfusion trigger was shown to be at least as effective as, and possibly superior to, a liberal transfusion strategy in critically ill patients, with the possible exception of patients with acute myocardial infarction and unstable angina [33]. Similarly, a restrictive strategy with Hb transfusion trigger of 7 or 8 g/dL was shown to be non-inferior to liberal transfusion strategy in postoperative orthopedic [34] and in cardiac surgery [35] patients. As a result of these and other similar studies, most clinical practice guidelines recommend restrictive RBC transfusion practices.

The rationale for restrictive transfusion strategy stems from the recognition that exposure to allogeneic blood is associated with many potential complications, including infection, mis-transfusion, ABO-incompatibility, transfusion reaction, transfusion-related acute lung injury (TRALI), transfusion-associated circulatory overload (TACO), elevated risk of cancer, and more [36]. Another major factor in the move to restrict transfusion is the significant cost that is involved, since the true cost, including processing, storage, viral testing, and other overhead costs, amounts to three to four times the acquisition cost [37]. For example, one study recently stated that even a 10 % reduction in RBC in a large US institution would result in more than $1,000,000 in blood acquisition cost savings [31].

In spite of the recommendations to use restrictive transfusion strategies, it seems that many clinicians still use a more liberal transfusion strategy, often termed the “10/30 rule” (Hb of 10 g/dl, hematocrit of 30 %) [31]. There may be a few reasons for this discrepancy. The first one, and probably the most common one, is the clinician’s fear of severe anemia especially when further bleeding is anticipated. In addition, preoperative anemia, even to a mild degree, has been recently shown to be independently associated with an increased risk of 30-day morbidity and mortality in patients undergoing major non-cardiac surgery [38]. A more forgiving attitude to perioperative blood transfusion may also be due to the practice of goal-directed therapy (GDT) which advocates maximizing cardiac output (CO) and DO_2_. For example, in the very recent POM-O study, in which achievement of preoperative DO_2_ values in the postoperative phase was attempted in high-risk surgical patients, the number of patients that received blood transfusion intra- and postoperatively in the GDT group was nearly double than the number in the control group [1]. Other reasons for not adhering to a restrictive transfusion regimen may include the growing perception that transfusion trigger in the individual patient is too complex and important to be guided by a single Hb value alone [39, 40]. In addition, the widely circulated recommendations of the Surviving Sepsis Campaign regarding blood transfusion in patients with septic shock include a recommendation for transfusion to maintain a hematocrit of more than 30 % in the presence of hypoperfusion in the first 6 h [41]. This recommendation, by the way, has not been supported by a recent large trial where a transfusion trigger of 7 g/dL, as compared with transfusion of 9 g/dL, led to fewer transfusions but similar mortality at 90 days in patients with septic shock [42].

The introduction of continuous non-invasive SpHb monitoring may be potentially helpful in preventing unnecessary transfusion as it may allow clinicians to more confidently manage patients at lower Hb levels with the knowledge that further drops into a critical anemic range will become readily apparent [43]. Thus, a stable or a rising SpHb trend may convince the clinician that additional transfusion may be unnecessary, especially when there is a delay in receiving laboratory hemoglobin values [30]. Indeed many decisions to transfuse, especially in the operating room, are not preceded by Hb measurement [31, 43]. Adding SpHb monitoring to standard of care blood management resulted in a significant decrease in blood utilization in high blood loss neurosurgery [44] and during elective orthopedic surgery [43].

Additionally, and of more potential importance, is the ability of a dropping SpHb trend to alert the clinician to the possible presence of undetected bleeding [30, 36, 43, 44]. This may be of special importance in obstetrics and in spinal surgery which have been found to be overrepresented in the recent report from the Anesthesia Closed Claims Project on massive hemorrhage [45]. Common to many of these cases that ended up in litigation were lack of timely diagnosis, lack of timely transfusion, and reoperation [45].

Importantly, SpHb monitoring is not yet as accurate as laboratory hemoglobin, and it is therefore not intended today as its replacement. Its value-added benefits should be considered as supplementing intermittent, delayed laboratory values with continuous, real-time visibility of whether Hb is stable, increasing, or decreasing [46]. The accuracy of SpHb measurement may, however, be improved by in vivo adjustment using reference Hb values [47]. In addition, the initial algorithm of the SpHb has been significantly improved and further validation studies are under way. While awaiting these promising developments, clinicians should be cautious when making clinical decisions based on individual values of the SpHb. Nevertheless, the information provided by changes in the trended SpHb may be helpful in guiding transfusion decisions.

## Non-invasive monitoring of fluid responsiveness

New pulse oximeter sensors offer the continuous non-invasive measurement of the plethysmographic variation index (PVI), which is a measure of the respiratory-induced variations in the plethysmographic waveform which, in mechanically ventilated patients and under certain conditions, may reflect fluid responsiveness (FR) [48, 49]. FR, in turn, is the degree by which the cardiac output CO responds to a modification of preload, and its assessment may facilitate decisions regarding fluid management, at times better than the CO itself [50, 51].

Indeed, the CO is a main determinant of DO_2_ and as such, should be monitored in severely ill patients. However, CO is not measured very often in clinical practice due to issues of availability, invasiveness, cost, inaccuracy, and lack of belief in its value. In addition, the CO has a few significant limitations. The optimal CO in an individual patient is difficult to determine, a ‘normal’ or even high CO does not preclude the presence of inadequate tissue perfusion, a low CO value does not tell us what to do (fluids? inotropes?). Finally, in and by itself, the CO does not predict FR [52]. Hence, in order to correctly interpret the CO we need to combine several variables before deciding whether CO is adequate and how it can be optimized in the most effective manner [53].

The actual monitoring of CO has increased significantly in recent years with the growing practice of perioperative GDT, which is largely based on CO maximization with fluids, with or without the addition of inotropes. This practice has usually resulted in larger amounts of fluids, usually colloids, being given to the patients receiving GDT [1, 54]. In spite of the earlier evidence and appeal of GDT that is based on CO maximization, many recent studies have failed to demonstrate its efficacy [1, 54, 55]. It may well be that GDT has frequently failed to improve outcome because it was based on CO (or stroke volume) maximization only, without taking into account FR, namely, predicting whether the patient is going to respond to fluids before the actual administration of fluids [56]. This problem has been inadvertently highlighted in a recent observational sub-study of the OPTIMISE trial [54], which included 100 of the 368 patients allocated to the CO-guided hemodynamic therapy algorithm [57]. According to the results of this study, which by the way was critical of the predictive accuracy of dynamic parameters, only 159 (28.6 %) of 556 fluid challenges were associated with an increase in the stroke volume. The significance of this report cannot be underestimated. If, in one of the most significant trials of GDT, more than 70 % of fluid challenges were administered to “non-responders”, there must be a fundamental problem with this approach. A much more logical approach was stated in a recent review, namely, that fluids should be administered only when patients require augmentation of their perfusion and are also volume-responsive [58].

The use of dynamic parameters, such as the systolic pressure variation (SPV), pulse pressure variation (PPV) and stroke volume variation (SVV), to assess FR in mechanically ventilated patients has become very prevalent in recent years [50, 51]. Basically, this approach is based on the assessment of the response of the left ventricular stroke volume (or any of its surrogates) to the preload-modifying effect of the mechanical breath, which serves as a repetitive hemodynamic challenge [50, 51]. The PVI is another dynamic parameter that can assess FR through the pulse oximeter sensor in a non-invasive manner and in the absence of an arterial line. The PVI is calculated as [(PI_max_ − PI_min_)/PI_max_] × 100, where PI_max_ and PI_min_ represent the maximal and the minimal values, respectively, of the plethysmographic perfusion index (PI, the ratio of non-pulsatile to pulsatile blood flow through the peripheral capillary bed) over one respiratory cycle [59, 60].

As with other dynamic parameters, the greater the PVI the more likely the patient will respond to fluid administration. A PVI >14 % before volume expansion was found to discriminate between ‘responders’ and ‘non-responders’ with 81 % sensitivity and 100 % specificity [61], and similar cut-off values were found by others as well. The PVI was shown to be similar to the PPV and SVV in its predictive ability of FR [49, 62]. Additionally, a higher PVI is correlated with a greater response of the CO to volume expansion [49, 61, 62].

The potential benefit of using the PVI to guide fluid management is demonstrated in 3 separate studies, in which intraoperative PVI-guided fluid management resulted in significantly less fluids being administered compared with the control standard therapy group [63–65]. In the most recent study, where PVI was used as part of an Enhanced Recovery protocol, fluids were administered only when the PVI exceeded 13 %, resulting in decreased intra-operative net fluid balance from 2733 to 848 ml [64]. These results stand in contradistinction to GDT studies where the intervention group traditionally received more fluids. They also support our earlier recommendation that FR should be assessed by dynamic parameters, when appropriate, before any fluids are administered [51]. Hence the PVI may prevent unnecessary and potentially damaging fluid overload as low PVI values mean that the patient is most probably not going to respond to fluids. At the same time, high PVI values may alert the clinician to the development of hypovolemia that may not be reflected by other commonly measured parameters [50, 51].

The PVI has the same limitations as all other dynamic parameters, the main ones being the presence of spontaneous ventilation, arrhythmias, too high or too low tidal volumes, and right heart failure [50]. Spontaneous ventilation does indeed preclude the use of the PVI for the prediction of FR. However, excessive large variations in the plethysmographic waveform during spontaneous ventilation should not be ignored as they may be an important sign of increased respiratory effort. A high PVI value during spontaneous ventilation, termed sPVI, has been recently shown to be a most sensitive sign of upper airway obstruction [66]. Like with all other dynamic parameters, a good predictive ability of FR necessitates a tidal volume of at least 7 ml/kg and will be reduced when lower tidal volumes are employed. In addition, the PVI may be less reliable than PPV and SVV for predicting FR in critically ill patients receiving norepinephrine [67]. One should bear in mind, however, that this monitoring modality should be reserved for patients for whom it is accurate and for whom no other dynamic parameter is available, or, in the words of Cannesson, “no high heels on the farm; no clogs to the opera” [68].

## The power of the multi-parametric approach

The most advanced version of the pulse oximeter offers, in addition to SpO_2_, new parameters such as the ORI, the SpHb and the PVI, which are all related to DO_2_. However, knowing the value of the DO_2_ may not be helpful without knowing the values of its individual components. On the other hand, discussing these parameters individually, as was done in this review, carries the risk of missing the power that a multi-parametric approach brings to cardiorespiratory monitoring. Each parameter that we measure has inherent limitations, confounding factors and inaccuracies. It is only by combining these parameters together that we can make best use of our monitors. The combination of SpO_2_ and the newly introduced ORI allows a better assessment of the oxygenation status in patients receiving oxygen, while the addition of capnography or respiratory rate monitor may better identify ventilatory problems. The SpHb can greatly facilitate patient blood management, and yet transfusion decisions should take into account other factors as well. For example, the combination of SpHb and the PVI may be especially useful as a low the level of the latter may help the clinician to withhold blood transfusion even if the SpHb is low. The PVI itself does predict FR and yet FR in and by itself is not an indication for fluid administration. The combination of all these (and other) parameters offers us a more holistic understanding of the pathophysiological status of the acutely ill patient. Such understanding is crucial for making the right decisions in a timely manner and for preventing the severe potential side-effects of our very potent therapies, namely fluids, blood and oxygen.
